# Correlation of mammographic density and serum calcium levels in patients with primary breast cancer

**DOI:** 10.1002/cam4.1066

**Published:** 2017-05-02

**Authors:** Carolin C. Hack, Martin J. Stoll, Sebastian M. Jud, Katharina Heusinger, Werner Adler, Lothar Haeberle, Thomas Ganslandt, Felix Heindl, Rüdiger Schulz‐Wendtland, Alexander Cavallaro, Michael Uder, Matthias W. Beckmann, Peter A. Fasching, Christian M. Bayer

**Affiliations:** ^1^ Department of Gynecology and ObstetricsErlangen University HospitalComprehensive Cancer Center Erlangen/European Metropolitan Area Nuremberg (CCC ER‐EMN)ErlangenGermany; ^2^ Institute of Biometry and EpidemiologyFriedrich Alexander University of Erlangen‐NurembergErlangenGermany; ^3^ Medical Center for Information and Communication TechnologyErlangen University HospitalErlangenGermany; ^4^ Institute of Diagnostic RadiologyErlangen University HospitalErlangenGermany; ^5^ Department of MedicineDivision of Hematology/OncologyDavid Geffen School of MedicineUniversity of California at Los AngelesLos AngelesCAUSA

**Keywords:** Body mass index, breast cancer prevention, breast density, calcium, mammographic density, menopausal status

## Abstract

Percentage mammographic breast density (PMD) is one of the most important risk factors for breast cancer (BC). Calcium, vitamin D, bisphosphonates, and denosumab have been considered and partly confirmed as factors potentially influencing the risk of BC. This retrospective observational study investigated the association between serum calcium level and PMD. A total of 982 BC patients identified in the research database at the University Breast Center for Franconia with unilateral BC, calcium and albumin values, and mammogram at the time of first diagnosis were included. PMD was assessed, using a semiautomated method by two readers. Linear regression analyses were conducted to investigate the impact on PMD of the parameters of serum calcium level adjusted for albumin level, and well‐known clinical predictors such as age, body mass index (BMI), menopausal status and confounder for serum calcium like season in which the BC was diagnosed. Increased calcium levels were associated with reduced PMD (*P* = 0.024). Furthermore, PMD was inversely associated with BMI (*P* < 0.001) and age (*P* < 0.001). There was also an association between PMD and menopausal status (*P* < 0.001). The goodness‐of‐fit of the regression model was moderate. This is the first study assessing the association between serum calcium level and PMD. An inverse association with adjusted serum calcium levels was observed. These findings add to previously published data relating to vitamin D, bisphosphonates, denosumab, and the RANK/RANKL signaling pathway in breast cancer risk and prevention.

## Introduction

Percentage mammographic breast density (PMD) is one of the most important risk factors for breast cancer (BC) 1, 2. Women with a high PMD have an up to fivefold increased risk for BC 3, 4, 5. It has been shown that a high PMD is associated with a greater relative risk for BC than family history or menstrual, hormonal, and reproductive risk factors, for example 6. Only age and *BRCA* mutation status have a higher relative risk for developing breast cancer than a PMD over 50% compared with a PMD lower than 10% 7, 8, 9, 10. It is thought that approximately one‐third of breast cancers may be explained by high PMD (American College of Radiology classification 3 and 4) 11.

Fibroglandular breast tissue appears radiopaque on mammography, whereas adipose breast tissue appears transparent or nondense 12. Studies have shown that the pathological correlate of PMD is related to proliferation with a stromal composition, rather than to epithelial changes 13, 14. PMD has a genetic predisposition 10, 15, 16 and is regulated by growth factors and hormones 17, 18.

A positive key feature of PMD in comparison with other established risk factors for breast cancer is its modifiability and dynamics. To date, several factors influencing PMD have been identified. PMD typically decreases with age, body mass index (BMI), which are independent risk factors for BC themselves 11, 19, 20, 21. Multiparity and breastfeeding are also associated with lower PMD 22, 23, and there is a significant reduction in PMD after the menopause 4, 24. Women with prolonged estrogen exposure, as in cases of late first pregnancy and early menarche, with hormone replacement therapy or alcohol consumption show higher degrees of PMD 25, 26, 27, 28.

Calcium, vitamin D, bisphosphonates, and denosumab have also been considered and partly confirmed as influencing factors for the risk of BC. An inverse dose–‐response association between calcium intake and breast cancer risk was described. A large meta‐analysis of eleven prospective studies showed that the overall risk reduction in breast cancer for high versus low intake of calcium was 0.92 (95% CI 0.85, 0.99) 29. Two recent meta‐analysis of prospective studies showed that overall vitamin D blood levels are associated with reduced breast cancer risk 30, 31. In several studies, treatment with bisphosphonates in the preventive situation showed an improvement in risk reduction. Recently, a meta‐analysis by the Early Breast Cancer Trialists’ Collaborative Group (EBCTCG) demonstrated a significant risk reduction for metastases, a reduction in local recurrences and mortality, and longer overall survival in postmenopausal breast cancer patients receiving adjuvant therapy with bisphosphonates 32. The analyses from the ZO‐FAST study and the ABCSG‐12 study also support the use of bisphosphonates in the adjuvant situation for BC prevention and risk reduction 33, 34.

Recent studies have provided a strong rationale that the RANK/RANKL signaling pathway, which controls amongst other things the regulation of calcium metabolism 35, is involved in mammary epithelial proliferation, carcinogenesis, and BC development 36, 37. RANKL inhibition — for example, using denosumab, a monoclonal antibody against the receptor activator of nuclear factor‐kappa B ligand (RANKL) — might therefore provide a novel approach to the prevention and treatment of BC 36, 37.

There are limited data concerning vitamin D and calcium in connection with reducing BC risk. Some studies have reported that calcium and vitamin D intake reduces PMD in premenopausal women, but not in postmenopausal women 38, 39, 40, 41, 42. A protective effect of calcium and vitamin D against breast cancer has also been considered 43, 44. The calcium metabolism thus appears to play a role in the process and in changes in breast tissue. It could therefore be hypothesized that calcium might have an influence on PMD and possibly on breast cancer risk.

The aim of this study was to examine the association between PMD and the serum calcium level in a large retrospective study of primary BC patients with high‐quality PMD measurements — specifically, whether serum calcium can be used to predict the PMD in addition to other, previously reported modifiers of PMD that correlate with a lower PMD, such as age, menopausal status, and BMI.

## Patients and Methods

### Study design

This study is a retrospective, single‐center, observational study in which the association of PMD and serum calcium levels was evaluated in patients with unilateral primary BC. It was conducted in accordance with the World Medical Association Declaration of Helsinki. Approval for the study was obtained from the ethical review committee in the Faculty of Medicine at Friedrich Alexander University of Erlangen–Nuremberg. All patients provided written informed consent after receiving detailed instructions and before inclusion in the NIS.

### Patient selection

The patients were selected from the BC database at the University Breast Center Franconia (Germany). All patients with invasive BC diagnosed between 2002 and 2010 at the University Breast Center Franconia were initially included in the database. A total of 5110 patients with invasive BC were documented in the database for the period 2002–2010. For the analysis presented here, patient datasets were excluded in the following order: missing calcium and albumin values around the date of the first diagnosis of breast cancer (±3 weeks; excluding 2134 patients with missing values for either calcium or albumin); and no mammograms taken and digitally stored around the time of diagnosis (not more than 3 months prior to diagnosis; excluding 1994 patients for whom the mammogram from the time of diagnosis was missing). The final dataset consisted of 982 patients at the University Breast Center Franconia with unilateral breast cancer and with calcium and albumin values and mammograms available from the time of first diagnosis. The patient characteristics of included and excluded patients are shown in Table S1. Both groups are similar.

### Clinical data

All patient characteristics and tumor characteristics were documented as part of the certification processes required by the German Cancer Society (*Deutsche Krebsgesellschaft*) and by the German Society for Breast Diseases (*Deutsche Gesellschaft für Senologie*) 45. Certification requires tumor characteristics, treatment data, some epidemiological data, histopathological characteristics, tumor treatments, and follow‐up data to be documented and audited annually. The BMI at the time of diagnosis was obtained from measurements in the hospital, which were carried out during treatment planning (i.e., before surgery or chemotherapy). The hormone receptor status of the tumor was assessed from the estrogen receptor and the progesterone receptor. If either of these receptors was expressed the patient was considered hormone receptor positive, otherwise hormone receptor negative. The serum calcium levels were corrected in adjustment to the serum albumin level. The time of diagnosis of breast cancer was also the time of measurement of the calcium level and albumin. The season was included as predictor, because there are seasonal variabilities of vitamin D and calcium levels. None of the patients was taking calcium or vitamin D or receiving bisphosphonates or denosumab.

### Mammographic density measures

The quantitative computer‐based threshold density assessments and breast area measurements were made by two different readers with explicit training in the method used. Each mammogram was read by both readers, independently of each other. Only the measurements for the contralateral healthy breast were used for the analysis. The assessment method has been described and validated previously elsewhere 46. Briefly, the images (screen‐film images and printouts of processed digital images) were digitized, using the CAD PRO Advantage film digitizer (VIDAR, Herndon, VA, USA), and for assessment of the density fraction, the reader used the Madena software program, Version X (Eye Physics, LLC, Los Alamitos, CA, USA) 46. All mammograms were read in random order by two different observers, who were unaware of any previous classifications or pathological findings. The average of the two observers’ values for percentage mammographic density (PMD) was used for this analysis, into which only measurements of contralateral craniocaudal (CC) images were included 47. In 6.4% of the readings there was a discrepancy in PMD measurements more than 20%. These cases were measured again not knowing the previous results. If the discrepancy still was >20% the patient was excluded.

### Statistical considerations

Linear regression analyses were conducted to investigate the impact on the percentage mammographic density (PMD) of the examination parameters of serum calcium level adjusted for albumin level (continuous), as well as several well‐known clinical predictors, such as age, continuous; body mass index (BMI), continuous and menopausal status, categorical. Season of diagnosis was included in the model because it could be a confounder for vitamin D and calcium levels.

Initially, a full linear regression model was set up with all of the predictors mentioned above and PMD as outcome variable. Then, backward stepwise variable selection in which serum calcium level, age, and BMI were kept was carried out to obtain the best model in accordance with the Akaike information criterion (the *final regression model*). The predictor serum calcium level was kept because it is the predictor of interest, the predictors age and BMI were kept because it is known that they are associated with PMD. The regression coefficients and *P* values for the predictor variables were estimated. The coefficient of determination, *R* ^2^ *,* was calculated to measure the goodness of fit.

Furthermore, the association between corrected serum calcium levels and PMD is shown, using a scatter plot that contains a regression line to indicate the trend of the association.

All of the tests were two‐sided, and a *P* value of <0.05 was regarded as statistically significant. Calculations were carried out, using the R system for statistical computing (version 3.2.2; R Development Core Team, Vienna, Austria, 2015).

## Results

### Patient characteristics

The analysis was conducted with complete datasets for 982 breast cancer patients, 2134 patients were excluded because of missing values, either calcium or albumin and 1994 patients were excluded due to missing mammograms. The PMD of included patients ranged from 3% to 93%. The patients’ average age was 59.2 (±12.3) years and their average BMI was 26.5 (±5.2); 24.6% (*n* = 242) of the patients were premenopausal and 75.36% (*n* = 740) were perimenopausal or postmenopausal. Excluded and included patients were similar (Table S1).

Serum calcium levels ranged from 1.7 to 3.0 mmol/L (reference range 2.20–2.65 mmol/L), and serum albumin levels from 14.4 to 54.5 g/L (reference range 33–55 g/L). The frequency of diagnosis did not depend on season. The patients’ and tumor characteristics are listed in Table 1.

**Table 1:** Patient characteristics and tumor characteristics, showing means and standard deviation (SD) for continuous characteristics and frequencies and percentages for categorical characteristics

Patient or tumor characteristic	Mean or *N*	SD or %
Calcium level adjusted to albumin (mmol/L)^a^	2.40	0.12
Body mass index (kg/m^2^)	26.46	5.20
Age (years)	59.21	12.33
Percentage mammographic density	36.73	19.89
Season of diagnosis of breast cancer		
Spring	250	25.46
Summer	242	24.64
Fall	253	25.76
Winter	237	24.13
Menopausal status		
Premenopausal (or after hysterectomy and <50 years)	242	24.64
Perimenopausal/postmenopausal (or after hysterectomy and age ≥50 years)	740	75.36
Pathological tumor size (pT)		
pT0^b^	1	0.10
pT1	593	60.39
pT2	262	26.68
pT3	45	4.58
pT4	30	3.05
Missing data	51	5.20
Pathological nodal status (pN)		
pN0	670	68.23
pN1	186	18.94
pN2	68	6.92
pN3	33	3.36
Missing data	25	2.55
Hormone receptor status		
Negative	179	18.23
Positive	763	77.70
Missing data	40	4.07
HER2		
Negative	778	79.23
Positive	162	16.50
Missing data	42	4.27
Grading (G)		
G1	166	16.90
G2	556	56.62
G3	203	20.67
Missing data	57	5.81

a Reference range for serum calcium: 2.2–2.65 mmol/L.

b pT0 = no evidence of primary tumor after primary systemic therapy.

### Prediction of PMD

The final linear regression model for predicting the impact of clinical predictors and serum calcium on the percentage mammographic density consisted of the predictors albumin adjusted serum calcium level, BMI, age, and menopausal status. Season of diagnosis did not remain in this model. The coefficients and *P* values of the final linear model are shown in Table 2.

**Table 2:** Final linear regression model for predicting the impact of clinical predictors on the percentage mammographic density (PMD).^a^ Regression coefficients with 95% confidence intervals (CIs) and *P* values are shown

	Coefficient (95% CI)	*P* value
Baseline	132.68 (109.89, 155.47)	<0.001
Calcium level adjusted to albumin	−11.37 (−21.23, −1.51)	0.024
Body mass index (kg/m^2^)	−1.63 (−1.82, −1.44)	<0.001
Age (years)	−0.37 (−0.48, −0.26)	<0.001
Menopausal status		
(reference), premenopausal	–	–
Perimenopausal/postmenopausal	−6.55 (−9.80, −3.30)	<0.001

a For example, for a 50‐year‐old perimenopausal women with a body mass index of 28 and calcium level of 2.5 mmol/L, the predicted percentage mammographic density is as follows: PMD = 132.68 + 2.5*x*(−11.37) + 50*x*(−0.37) + 28x(−1.63) + 1*x*(−6.55) = 33.57.

Age and BMI were both significantly and inversely associated with PMD. There was also an association between PMD and menopausal status. Older women had lower mammographic densities (*P* < 0.001) as well as postmenopausal women (*P* < 0.001). A higher BMI was associated with a lower PMD as well (*P* < 0.001).

With regard to calcium a higher calcium was significantly associated with lower PMD (*P *=* *0.024). Every increase in calcium serum levels by 0.1 mmol/L would decrease the predicted PMD in the linear regression model by 1.1%.

A “high‐density patient” can thus be defined by this linear regression model as a young, slim woman with a low calcium level, while a “low‐density patient” is an elderly, obese woman with a high calcium level. Goodness of fit of the regression model was moderate (*R* ^2^: 0.39).

**Figure 1:**
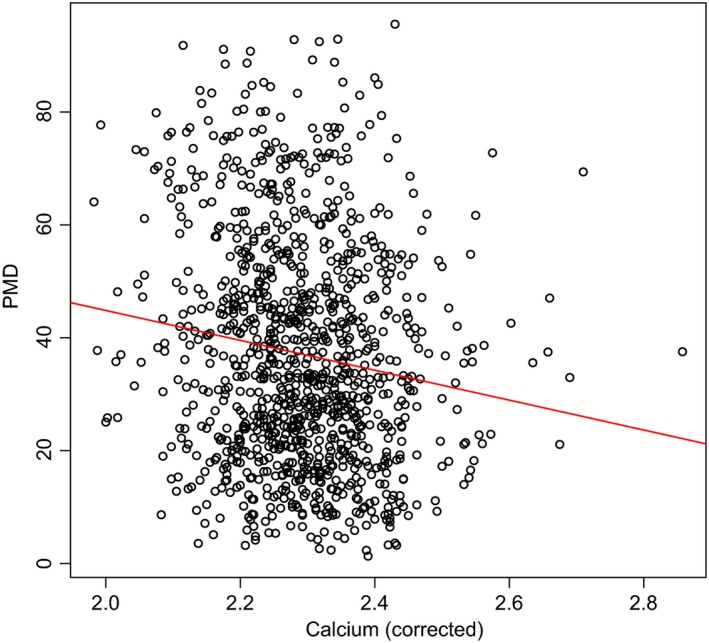
The scatter plot shows a weak inverse association (*r* = −0.14) between the corrected calcium levels (in mmol/L) and percentage mammographic density.

The association between calcium and PMD is also pictured in Figure 1.

## Discussion

This retrospective noninterventional study shows that PMD, measured by computer‐assisted assessment, is significantly associated in BC patients with serum calcium level, BMI, age, and menopausal status. There was an inverse association between calcium and PMD.

The results, showing that PMD decreases with increasing age, BMI, and menopausal status, were expected. There is considerable evidence that PMD is negatively associated with BMI 22, 48, 49. High BMI reduces PMD by increasing the fat proportion in the breast 20, 21, 50. Several studies have demonstrated that PMD typically decreases with age 12, 25, 47. The reduction in PMD with increasing age reflects the reduction in glandular tissue and the accompanying increase in fat that occurs with aging. As expected, the present results relating to menopausal status are consistent with the data from other previous studies. The evidence shows that PMD decreases during and after menopause 4, 24.

A weak inverse association was also found between serum calcium levels, adjusted to serum albumin, and PMD in patients with primary breast cancer. An increase in the calcium level correlated with a decrease in PMD. To date, there have been no similar studies investigating the correlation between the serum calcium level, adjusted to albumin, and PMD.

The reduction in PMD associated with calcium intake during the premenopause has been consistently confirmed in several studies 20, 38, 39, 41, 42, 44. The results in the postmenopause have so far been inconsistent 39, 42, 51, 52. Two studies reported a reduction in PMD with vitamin D and calcium intake, at least partially 40, but several studies in a variety of other populations have not observed a relationship between PMD and intake of either vitamin D 20, 39, 51, 53 or calcium 20, 39, 51, 53.

With regard to the correlation between 25‐hydroxyvitamin D (25(OH)D) levels and/or serum calcium level and PMD, most previous studies have only taken into account the 25‐hydroxyvitamin D (25(OH)D) level, without the serum calcium level. The 25(OH)D level has been associated with breast cancer risk 54, 55, but data for PMD and 25(OH)D have shown mostly negative results 56, 57, 58, 59, 60. Only one small study including 238 postmenopausal healthy women has examined the influence of 25(OH)D and the calcium level on PMD 61, and it did not find a significant association 61. This study is thus the first study to assess the association between the serum calcium level, adjusted to serum albumin, and PMD in patients with primary BC.

Some evidence has recently been found that RANKL mediates progestin‐induced mammary epithelial proliferation, carcinogenesis and BC development 36, 37. The RANK/RANKL signaling pathway controls the development and activation of osteoclasts, the regulation of calcium metabolism, and the formation of lactating mammary gland tissue during pregnancy 35. In contrast, genetic inactivation of the RANKL receptor RANK in mammary‐gland epithelial cells and deletion of RANK from the mammary epithelium prevent hormone replacement therapy‐induced epithelial proliferation and the incidence of BC. All of these data suggest that RANKL inhibition by an anti‐RANKL antibody such as denosumab could be used in the future for prevention and treatment of BC 36, 37. Further studies on the antitumor effects of denosumab in adjuvant breast cancer care are in progress.

Against this background, the present results are of particular interest. The exact mechanism through which low serum calcium levels lead to a higher PMD, which is associated with an increased risk for BC, is not yet known. However, in view of recent research on RANKL and BC development, as well as the regulation of the calcium metabolism by RANKL, it may be hypothesized that the RANK/RANKL signaling pathway plays a special role in the complex correlation between serum calcium levels and PMD. This study might therefore support a rationale for establishing denosumab as a preventive treatment against BC.

The study has several strengths and limitations. The BC patients included were hospital‐based and were not recruited from a population‐based screening program. Breast density is thought to contribute to a higher likelihood of tumors being missed during early detection methods for BC. It might therefore be possible that the patients had a higher PMD than that in the general population. The large amount of missing values could lead to a selection bias. But on the other hand, the collective was well characterized and published in others studies before. Another study limitation is the lack of information on the correlation between serum calcium and PMD in healthy women not diagnosed with breast cancer. It could be that the presence of breast cancer has influenced the association of serum calcium and PMD. Not all well‐known predictors (e.g. present/previous hormone therapy, smoking or alcohol intake) of PMD are included in the model. The correlation between a higher calcium and lower PMD was significant, but the goodness of fit of the regression model was moderate. Our regression analyses showed that age, BMI, menopausal status, and calcium levels are associated with PMD. A precise quantification of the association, however, was not possible due to moderate goodness of fit of the regression model.

The strengths of the study are the semiautomated method of quantifying mammographic density, with two readers for all images and a mean value for PMD being used. This may reduce measurement inaccuracies. Both semiautomated assessments and automated volumetric density showed similar results 62, 63, 64, 65. Especially in images with low quality, automated assessment programs have higher (about 10%) failure rate of valid density values compared to semiautomated programs. Another strength is the use of only incident BC cases, avoiding the risk of bias in the selection of patients due to effects of tumor and tumor therapy on calcium metabolism. The serum calcium level was corrected with adjustment to the serum albumin level (about 50% of calcium is linked to albumin in serum), which provides more detailed information about the real serum calcium value. In addition, the large sample size, with more than 900 patients, makes the detection of smaller effects more likely, although the study did not identify any. The assessments of the serum calcium level and serum albumin level were carried out in the same central laboratory, promoting homogeneity in the assessment of these markers. The study highlights a highly topical matter that is currently the subject of several research projects and recent publications.

## Conclusions

An inverse association was observed between serum calcium levels, adjusted to serum albumin levels, and percentage mammographic density in patients with primary breast cancer. These findings, along with previously published research results, provide a strong rationale suggesting that the calcium metabolism and related pathways, including the RANK/RANKL signaling pathway, may play a role in the mechanism involved. Future research investigating BC prevention should specifically address the calcium and RANK/RANKL pathway.

## Conflict of Interest

PAF has carried out research for Novartis and Amgen. All of the other authors declare that they do not have any conflicts of interest.

## Supporting information


**Table S1.** Patient characteristics and tumor characteristics of included and excluded patients, showing means for continuous characteristics and percentages for categorical characteristics.
