# Supplementary material for: Correlation of mammographic density and serum calcium levels in patients with primary breast cancer
Source: Cancer Med. 2017 May 2;6(6):1473–81. doi: 10.1002/cam4.1066 (PMC5463083; doi:10.1002/cam4.1066)
Supplement: Supplementary file 1 — Table S1. Patient characteristics and tumor characteristics of included and excluded patients, showing means for continuous characteristics and percentages for categorical characteristics. [file CAM4-6-1473-s001.docx]

**Supplementary Table 1**: Patient characteristics and tumor characteristics of included and excluded patients, showing means for continuous characteristics and percentages for categorical characteristics

| Characteristic (continuous) | Included pts. | Excluded pts. |
| --- | --- | --- |
| n | 982 | 4128 |
| Body mass index (kg/m^2^) | 26.46 | 25.28 |
| Age (years) | 59.21 | 58.31 |
| Characteristic (categorical) |  |  |
| Menopausal status |  |  |
| Premenopausal (or after hysterectomy and age < 50 years) | 24.6 | 25.3 |
| Perimenopausal/postmenopausal (or after hysterectomy and age ≥ 50 years) | 75.3 | 74.7 |
| Pathological tumor size (pT)  pT0*  pT1  pT2  pT3  pT4  missing data | 0.1  60.3  26.6  4.5  3.0  5.2 | 2.7  57.2  27.2  4.2  1.7  7.0 |
| Pathological nodal status (pN)  pN0  pN1  pN2  pN3  missing data | 68.2  18.9  6.9  3.3  2.5 | 71.7  16.2  5.5  3.4  3.2 |

* pT0 = no evidence of primary tumor after primary systemic therapy
